# The Light and Shadow of Senescence and Inflammation in Cardiovascular Pathology and Regenerative Medicine

**DOI:** 10.1155/2017/7953486

**Published:** 2017-10-08

**Authors:** Laura Iop, Eleonora Dal Sasso, Leonardo Schirone, Maurizio Forte, Mariangela Peruzzi, Elena Cavarretta, Silvia Palmerio, Gino Gerosa, Sebastiano Sciarretta, Giacomo Frati

**Affiliations:** ^1^Cardiovascular Regenerative Medicine Group, Department of Cardiac, Thoracic and Vascular Surgery, University of Padua and Venetian Institute of Molecular Medicine, Padua, Italy; ^2^Department of Medico-Surgical Sciences and Biotechnologies, Sapienza University of Rome, Latina, Italy; ^3^IRCCS Neuromed, Pozzilli, Italy

## Abstract

Recent epidemiologic studies evidence a dramatic increase of cardiovascular diseases, especially associated with the aging of the world population. During aging, the progressive impairment of the cardiovascular functions results from the compromised tissue abilities to protect the heart against stress. At the molecular level, in fact, a gradual weakening of the cellular processes regulating cardiovascular homeostasis occurs in aging cells. Atherosclerosis and heart failure are particularly correlated with aging-related cardiovascular senescence, that is, the inability of cells to progress in the mitotic program until completion of cytokinesis. In this review, we explore the intrinsic and extrinsic causes of cellular senescence and their role in the onset of these cardiovascular pathologies. Additionally, we dissect the effects of aging on the cardiac endogenous and exogenous reservoirs of stem cells. Finally, we offer an overview on the strategies of regenerative medicine that have been advanced in the quest for heart rejuvenation.

## 1. Introduction

Cardiovascular diseases affect millions of patients worldwide and are the primary cause of mortality and comorbidities. Thanks to the incredible technological progress realized in the post second war period (1945–1964) in the Western countries, a whole generation of people started to benefit increased wealth and ameliorated life quality and expectancy [1].

For this generation and those to come, the prolonged lifespan is associated with a dramatic increase of cardiovascular diseases, mainly related to aging. This is at least partly due to a progressive impairment of the cellular processes regulating cardiac and vascular homeostasis, finally leading to the development of cardiovascular pathologies. In fact, the molecular mechanisms that protect the heart against stress are downregulated by aging, making the myocardium more susceptible to injury. Pathologies, as atherosclerosis, cardiac fibrosis, and cardiomyopathy, are often linked to the failure of cardiovascular tissue cells to reenter the cell cycle, namely, senescence, due to endogenous or exogenous causes.

This review will focus on the cardiovascular pathologies correlated to senescence, the effect of aging on the cardiac endogenous resources of stem cells, and the potential strategies of regenerative medicine to be applied to maintain the heart younger and healthier.

## 2. Positive and Negative Effects of Senescence on Cardiovascular Disease Onset and Progression

Cellular senescence has been for long time an underestimated biological process, even after its discovery in 1961 by Hayflick and Moorhead [2]. As demonstrated by the growing body of literature in the latest years, senescence is an important function involved in the maintenance of tissue homeostasis, as well as the more extensively studied apoptosis.

Mitotic cells might undergo senescence by failing to replicate. Unlike the quiescence state, in which cells are reversibly dormant, senescence takes place in cells with an active metabolism that had entered the cell cycle but got stopped at G1 phase by the action of specific inhibitors (as reviewed in [3]).

While it is commonly accepted as an aging-related phenomenon, senescence might happen also during the embryonic development with the biological meaning of replacing transient structures or specific cell types with other ones [3–5]. This is partly conflicting with the hypothesis of antagonistic pleiotropy as an evolutionary justification for senescence. In fact, it was George J. Williams to observe as first that it could be described as an adaptive event during evolution, since some genetic traits demonstrated to be beneficial in the initial life stages, but disadvantageous in the elderlies [6].

Senescence in adult tissues can be classified in two main subcategories, based on the underlying molecular mechanism: replicative (or intrinsic), caused by telomere shortening, and stress-induced, in response to reactive oxygen species (ROS) and/or oncogenes [7–9].

Activation of the cellular senescence genetic program prompts a series of molecular changes, mostly affecting cell cycle, extracellular matrix (ECM), secretion of growth factors, and inflammatory mediators.

A more detailed description of the triggering stimuli and molecular pathways involved in the pathogenesis of several cardiovascular diseases will be provided below.

Senescent cells might be easily recognized in culture by typical morphological features, that is, flatness, enlargement, vacuolization, multinucleation, and progressively reduced proliferation.

Hallmarks of cellular senescence are represented by lysosomal immunodetection of *β*-galactosidase activity (also known as senescence-associated *β*-galactosidase activity), lipofuscin rendered evident after Sudan black B staining, downregulation of proliferation markers, for example, phosphohistone 3 and Ki-67, reduced incorporation of 5-bromodeoxyuridin, identification of heterochromatin foci, and increased expression of senescence mediators (e.g., p15, p16, p21, p27, and ARF) and metalloproteinases. As previously mentioned, a further phenotypic characteristic of senescent cells is their secretion of several specific proinflammatory cytokines and growth factors (e.g., IL-6, IL-8, and TGF-*β*) [10]. Endowed with this senescence-associated secretory phenotype (SASP), cells exert a paracrine effect on neighboring cells, as well as an autocrine conditioning [11].

Consequently, the role of senescence in a variety of pathologies has been increasingly studied in the last years. Among cardiovascular diseases, atherosclerosis and heart failure are those whose progression has been shown to be most deeply associated with cellular senescence.

### 2.1. Atherosclerotic Lesions

Senescence-associated alterations are commonly observed in vascular cells harvested from atherosclerotic regions [12–16]. Atherosclerosis is a chronic inflammatory vascular disease that evolves from an initial dysfunction of the intimal endothelial layer to a progressive development of advanced atherogenic plaques [17].

High blood cholesterol levels, inflammatory cytokines, growth factors, angiotensin II, and hyperglycemia, with its associated advanced glycation end products (AGEs), are powerful extrinsic stimuli for vascular cell senescence [17]. Telomere shortening and activation of the Ras pathway are regarded as intrinsic vascular senescence triggers [17].

In addition, mitochondria have been revealed to play an important role in the development of atherosclerotic lesions. In fact, mitochondrial dysfunction, mtDNA mutations, and/or release of mitochondria-specific ROS in both senescent endothelial and smooth muscle cells are considered as further putative causes of atherogenesis [18].

As recently reviewed [19, 20], the molecular basis for aging of vascular cells involves several genes (as Klotho) and proteins (e.g., Sirtuins, progerin, JunD, p66^shc^, and *β*-amyloid peptides).

Replicative senescence in vascular cells activates molecular pathways similar to those triggered after DNA damages caused by external agents. The stress-induced typology is also frequently found in endothelial and smooth muscle cells from atherosclerotic tissues. A variety of pathways are interested in vascular DNA damage-induced cellular senescence, most converging on the activation of cyclin-dependent kinase inhibitors (p15, p16, p21, p27, and/or p53). The mediators of cell cycle progression are conversely downregulated, for example, derepressing the gene encoding for cyclin-dependent kinase inhibitor 2A. This prevents cyclin-dependent kinases (1, 2, 4, and 6) and cyclins (A, E, and D) to downregulate tumor suppressor RB. Consequently, the vascular cell is arrested at the Gap 1 phase of the mitotic replication cycle (as reviewed in [3]).

In addition to mentioned DNA damage-related pathways, activation of nuclear factor NF-*κ*B and CCAAT/enhancer-binding protein-*β* pathways is required by the senescent vascular cells to activate SASP program, which includes the secretion of IL-6, IL-8, chemokines, and activators of macrophages and monocytes (MCP, MIP, TNF-*α*, TGF-*β*, and GM-CSF) as well as ECM proteases [3, 21]. Interestingly, smooth muscle cells secrete also procalcific factors, such as RUNX-2, alkaline phosphatase, collagen I, matrix GLA protein, and BMP-2 [12, 22].

The proteolytic secretome of vascular smooth muscle cells, together with ROS and the enzymes released by recruited macrophages, contributes to undermining plaque stability in the atherosclerotic cap region through ECM remodeling [23].

Moreover, also endothelial nitric oxide synthase (eNOS) and prostacyclin pathways result impaired in the senescent endothelial cells from atherogenic regions [14, 24–26].

Growing evidence suggests a crosstalk between nitric oxide (NO) signaling and the occurrence of oxidative stress in the onset and progression of age-related vascular diseases, such as hypertension, heart failure, ischemia, and stroke. Consequently, NO is being considered as an emerging molecular target for the development of new therapeutic strategies for cardio- and cerebrovascular pathologies, particularly in aged patients. Accordingly, nowadays, several natural-derived compounds or pharmacological inhibitors are being proposed as modulators of NO-mediated pathways in this population. Curiously, vascular senescence is thought to have beneficial effects in the evolution of atherosclerosis. In particular, polymorphism analyses in humans and in atherosclerotic murine models revealed that lower levels of cyclin-dependent kinase inhibitors, especially CDKN1 and CDKNA2, are associated with increased propensity to develop atherogenic lesions [27, 28]. In other genome-wide associated studies, a link between aortic aneurysms and the polymorphisms for CDKNA2 and CDKNB2 has been shown [29].

On the other hand, other studies are supportive of a view of pathologic deterioration induced by senescent vascular cells. In fact, endothelial cells with an activated senescence program secrete high levels of ICAM-1, PAI-1, and IL-1*α* and show a reduced ability to metabolize lipids [30–33], which is likely to aggravate atherosclerosis.

It has been recently demonstrated that endothelial SASP, and in particular its microvesicular component, is able to stimulate the onset of calcification through the overexpression of bone-related proteins, for example, annexins and BMPs, and the increase of Ca^2+^ content too [34].

Typical hallmarks, triggers, and effects of vascular senescence in atherosclerosis have been summarized in Table 1.

### 2.2. Heart Failure

A potentially predictive biomarker of cardiovascular senescence is leukocyte telomere length. Remarkably, its shortening is associated with several cardiovascular diseases, among which are atherosclerosis, aortic valve stenosis, and thrombogenesis. Moreover, it is also linked to the main risk factors for these pathologies, such as hypercholesterolemia and hypertension. Leukocyte telomere length correlates inversely with plaque progression, but also heart failure (HF), a severe chronic condition characterized by dilation and decreased thickness of the ventricular wall. HF is the final stage of all cardiovascular diseases and results in a progressive weakening of the global cardiac function, which is related to the dysfunctional hypertrophic and apoptotic state of the terminally differentiated cardiac myocytes [33, 35–40].

Besides apoptosis, autophagy is as well a crucial mechanism for maintaining cellular homeostasis during aging, since it ensures the removal of dysfunctional organelles and misfolded proteins that dramatically increase in aged organs. Generally, elderly patients have reduced levels of autophagy, probably due to the elevation of oxidative stress. In this regard, it has been demonstrated that cardiac-specific deletion of Atg5 in mice associates with early signs of senescence, accumulation of dysfunctional mitochondria, disorganization of sarcomere structure, and age-related cardiomyopathy [41]. Similarly, mice deficient in Parkin gradually showed a decrease in cardiac function and survival [42]. Coherently, experimental evidence suggests that the modulation of autophagy may slow down or prevent cardiac aging. For example, stimulation of mitophagy (i.e., autophagy of mitochondria) with spermidine, a natural polyamine, was found to preserve cardiac function in old mice [43, 44]. Similarly, inhibition of miR-22, whose expression was reported to be elevated in the aged myocardium in parallel to decreased autophagic activity, rescues autophagy and improves cardiac function in old mice [45, 46].

Alterations of the inflammatory, endothelial, and myogenic phenotype of cardiac cells are also observed in senescence events occurring in the failing heart. In particular, most of these changes are related to the aging-dependent manifestation of mutations in genes involved in the calcium cycling and signaling. The activity of sarcoplasmic reticulum calcium adenosine triphosphatase, that is, SERCA2, and calsequestrin is drastically decreased during aging [47, 48], due to the lower level of expression of these proteins. Similarly, also Ica, that is, the L-type calcium current, is reduced and often completely inactivated. In this condition, the sarcoplasmic reticulum has a considerably diminished calcium concentration and, consequently, calcium transient amplitude and propagation result were impaired.

Nevertheless, also other pathways involved in heart rate modulation might be compromised by aging, for example, as cardiac sympathetic innervation. In fact, the catecholamine uptake is reduced in senescent cardiac neuronal cells as well as it is the response to isoproterenol [49]. In particular, the decreased epinephrine reuptake is physiologically compensated by the reduction of the arterial baroreflex response [49]. Moreover, noradrenalin transporter is downregulated in aging, causing an impairment in the neurotransmitter reuptake and a consequent reduction of its positive inotropic effect [50].

The human K^+^ channel ether-à-go-go gene (hERG) might also be interested by age-dependent mutations causing rhythm instabilities, as well as disorganization of the structure of the sarcomeric structure and myofibrillary proteins, as evidenced in a mouse model of a mutated hERG homolog [51].

However, all these aging-related impairments increase the vulnerability of the single cells and hence of the whole myocardium to develop arrhythmic events. In fact, these occurrences are typical of elderly patients, which are therefore submitted to ICD implantation.

In addition to cardiac myocytes and neuronal cells, senescence affects also the vascular compartment, for example, compromising coronary microcirculation.

Cardiomyopathy-induced HF has very similar signs to those described for atherosclerosis. Furthermore, it has been shown that aging downregulates the mitochondrial nicotinamide adenine nucleotide histone deacetylase (SIRT) 3, which is associated with pericyte loss and endothelial dysfunction, further exacerbated in the case of concomitant diabetic condition [52].

In the presence of risk factors, such as obesity, aging-related microvascular rarefaction is correlated to adipose tissue activation of the ADAM 17/TACE gene, encoding for a metalloproteinase that is able to cleave out the active TNF-*α* polypeptide from pro-TNF-*α*. ADAM-17/TACE overexpression is also facilitated by the loss of negative regulation by caveolin-1, analogously decreased in aged obese patients [53].

In acute settings of cardiac ischemia, the activation of the senescence-associated genetic program in recruited fibroblasts can be considered a protective mechanism from endothelin-1-mediated cardiac fibrosis [54, 55].

Table 2 offers a schematic overview on the classic signs, causes, and consequences of senescence in heart failure settings.

A detailed description of age-related events that irreversibly compromise heart functionality is provided in these suggested review articles [3, 20, 56].

Moreover, other cardiovascular pathologies, as aneurysms and peripheral artery disease, have a strong correlation with the onset of senescence in the cellular and ECM components of interested tissues and might be implicated in the genesis of heart failure [57–62].

## 3. Tissue Development, Regeneration, and Aging in the Heart

During ontogenesis, the heart originates from the mesodermal tissue of the embryonic cardiogenic plate. From this, the primitive cardiac tube develops, loops and twists, and finally forms the atria, ventricles, and outflow tracts [63].

More accurately, two heart fields in strict vicinity and relation constitute the primitive cardiac tissue. In particular, the cellular elements of the cardiac crescent, or anterior heart field, will mainly develop the left ventricle and the atria, while the ones of the second heart field will mainly colonize the right ventricle and the outflow tract, with small contribution to the atria [64]. Indeed, the cells contributing to the development of the heart originate from several sources. As recently demonstrated, Isl-1 progenitors will give rise to the atria and outflow tract by following different specializations. The expression in Isl-1 progenitors of the cardiac-specific homeobox Nkx2.5 and of the patterning gene Wnt-2 is associated with the differentiation towards the atrial phenotype. For the development of the outflow tract and right ventricle, the same progenitors acquire a different phenotype, expressing Nkx2.5, the myocyte-specific gene Mef2-c, the transcription factor gene T-box 1, and the fibroblast growth factor genes Fgf-8/10. Other Isl-1 cells, coexpressing the early endothelial marker Flk-1, are more involved in the formation of the endothelial lining in the endocardial layer or in the vessels. In the immediate postnatal life, Isl-1 cells and cardioblasts progressively differentiate into mature cardiomyocytes [64, 65].

Additional cell types with immature and plastic characteristics have been identified in the heart. In particular, cardiac stem cells and progenitors have been detected and isolated from pediatric and adult heart tissues. These cells express a typical hematopoietic marker, that is, c-kit, in combination with other proteins and transcription factors, for example, the hematopoietic Sca1, CD34, and/or the more cardiac-committed Mef2-c and GATA-4 or the endothelial CD31. Cardiac stem cells have been shown to possibly originate all the lineages of the heart [66–70].

Further cells of particular interest in the heart tissues are the epicardial stem cells. As by their name, these cells can be retrieved in the epicardium and are identified by the expression of the Wilms tumor protein, codified by the gene Wt1 [71].

Exogenous cells with plastic features can participate in the organ development also in other phases. Neural crest cellular elements contribute to the construction of the distal outflow tract, working myocardium, glia, and nervous tissue during late gestation [72]. Additionally, endothelial progenitor cells are involved in the formation of new vessels in adult life [73].

Despite the existence of several cardiac stem cells and progenitors, the heart is not able to undergo a sustained self-renewal, especially after extensive damage. This property is related to its primary function, that is, pumping the blood throughout the whole body, which consumes massive amount of energy resources.

In the final stages of gestation, neonatal cardiomyocytes withdraw from reentering the cell cycle. Indeed, in the immediate postnatal period, cells undergo an incomplete mitosis and can be seen in the myocardium having dual or multiple nuclei, due to the inability to progress in karyo- or cytokinesis [74, 75]. In fact, they modify their growth modality from hyperplastic to hypertrophic and will not reenter the cell cycle. Actually, Ahuja et al. demonstrated that a preparatory dedifferentiation process, consisting of cycles of myofibrillary disassembly and reassembly, is necessary for a cardiomyocyte to divide [76]. However, a study of genetic cell fate mapping by Zhang et al.'s group revealed that mouse mature cardiomyocytes are able *in vitro* to undergo dedifferentiation-proliferation and show multipotent capacities [68].

Cardiac stem cells maintain their plastic ability during adult life, but remain quiescent in special microenvironments, namely, the cardiac niches, and are reactivated to replace the physiological loss of cardiomyocytes. However, the reactivation of the cell cycle only in cardiac stem cells is not sufficient to face the large loss of cardiac myocytes that occurs after an ischemic attack.

The sustained metabolic conversion of the heart is emblematic of mammalian species, but not of other vertebrates. Salamanders, axolotls, and zebrafish respond to heart injury inducing a blastema tissue, as part of an epimorphic regeneration [77]. This program is realized through the cell-cycle activation in mature cardiomyocytes, the degradation of the ECM, and a relatively low inflammatory response, mediated by a deficient adaptive immunity [78], in a tissue particularly poor of fibroblasts [79]. Conversely, mammalians have a higher fibroblast/cardiomyocyte ratio; thus, the upregulation of genes involved in ECM synthesis and a strong immunoinflammatory response dictate the outcome of the damage response towards a scar formation.

## 4. Senescence Events in the Endogenous Stem Reservoirs of the Heart and in Cardiac Cell Therapy

From their discovery, cardiac stem cells have been studied intensively to develop cardiac cell therapies for treating organ failure. Cardiac stem cells can be cultured *in vitro* through the generation of cardiospheres (CS) and then expanded serially as cardiosphere-derived cells (CDCs) for clinical application [66–69]. As with the other differentiated cells of the heart, also cardiac stem cells might undergo senescence, showing reduced telomere length and telomerase activity, expression of p16^INKA^ and p21^CIP^, and an IL-6- and IGFBP7-enriched SASP [80].

The molecular mechanism for the onset of senescence in these cells is still not completely elucidated. The cardiac niche is rich in fibronectin and thus should represent a protective environment, which, evidently, does not exclude stem cell conditioning by aging-inducing triggers. In addition, also the niche ECM is a potential target of aging.

Independently from their localization, senescent stem cells tend to accumulate during aging. Nakamura et al. have shown that an increased expression of the Wnt inhibitor Sfrp1, together with higher levels of p16 and a peculiar SASP, is typical of human CDCs of old subjects (65–83 years), in comparison to those derived from younger ones (2–65 years) [81]. A recent study by Piegari et al. evidenced that the antitumoral drug doxorubicin exerts its cardiotoxicity also in cardiac stem cells by inducing cardiac stem cell senescence. In fact, telomere shortening, impaired migration, and differentiation were demonstrated in the anthracycline-treated human cells [82]. In addition, their number, self-renewing, and clonogenicity abilities decrease during age-related cardiovascular pathologies, compromising heart function [35, 80, 83–85]. Interestingly, Wu et al. recently demonstrated in a mouse model that although aged animals in physiological settings possess a remarkable number of cardiac stem cells, these ones result dysfunctional in several biological activities, as the metabolism of vitamins and tyrosine, the circadian rhythm, and the complement and coagulation cascades. Consequently, cell proliferation, multipotency, and differentiation abilities are impaired [86].

Besides anthracycline-induced damage, a strong body of evidence suggests that other epigenetic modifications are introduced in cardiac stem cells undergoing senescence [87].

Apart from resident stem cells of the heart, senescence can affect also exogenous reservoirs, which are used for physiological and pathological heart remodeling and have been studied to develop new stem cell-based therapies. Hematopoietic stem cells (HSC) have, in fact, been widely applied for this aim, since they are relatively easy to select through a consolidated profile of surface differentiation markers used for cytofluorimetric cell sorting [88]. These cells reside in the bone marrow and can be mobilized in response to a variety of signals [89]. Induced quiescence, overexpression of Mdr1 and Abcg2 transporters, glycolysis-mediated ATP generation, telomere shortening, accumulated mitochondrial mutations, and reduced ROS production collectively turn these cells into a senescent state, without sustained abilities of self-renewal and differentiation [90–93]. Intriguingly, although a high number of HSCs is retrieved in the elderly heart, these undergo a drastic lowering of clonal diversity and switch towards the myeloid lineage, consequently impairing their regenerative abilities [94]. Early committed, mobilized HSCs, that is, endothelial progenitor cells (EPCs), might also display senescent features, for example, intensified ROS production prompted by overexpressed angiotensin II and increased induction of apoptosis, which can be both potentially associated with reduced levels of SDF-1 [95, 96].

In support of HSC performance, mesenchymal stem cells (MSCs), which were originally thought to reside in the bone marrow alone, can be found in a variety of different tissues and have been widely studied to perform cell therapies. For example, the control of adipose stromal cell (ASC) fate by epigenetic regulators might be an interesting tool to boost both cardiac commitment and regenerative capacities of these cells. Aged MSCs show a classic senescent phenotype and demonstrate reduced migration capacity, decreased plasticity [97], and alterations in their immunoregulatory abilities, that is, one of the most striking properties of these cells [88, 98–100].

Table 3 presents a summary of the altered characteristics of CSCs, CDCs, HSCs, EPCs, and MSCs during cell senescence.

## 5. Rejuvenation Biotechnology: Antisenescence Regenerative Therapies and Disease Modeling

Even though senescence was initially depicted as a beneficial program activated by the organism to eliminate aged, dysfunctional mutated cells, scientists are still debating its true biological significance [3, 80, 101, 102].

Cell clearance is the last and most critical stage of senescence progression. As mentioned before, aged cardiovascular tissues are incredibly enriched in differentiated as well as stem/progenitor senescent cells. Moreover, cell clearance has been related to regression of several diseases, as neurodegenerative disorders and cancer [103, 104].

It has been shown that in senescent cancer tissues, cell clearance is impaired due to mutation in cell cycle checkpoint-related genes, for example, p53, with severe deleterious effects on tumor progression [105].

Indeed, it is fascinating that evolutionarily inferior organisms, as the unicellular ciliate protozoa and the multicellular basal metazoans, sponges, cnidarians, and flatworms, do not show senescence and are able to remain in a quasi-immortal state [106], firstly defined by Finch in 1994 as negligible senescence [107]. A few years later, de Grey et al. introduced the concept of engineered negligible senescence, as to encompass all the biomedical strategies applied to reach a body rejuvenation in complex organisms, as the higher bilaterians [108].

In the quest of heart immortality, several therapeutic approaches have been explored, such as new drug generations, cardiac cell therapies, and tissue engineering strategies (Table 4).

Naturally inspired drugs have been formulated by the direct observation of the molecular pathways involved in senescence. Demaria et al. demonstrated that the administration of recombinant platelet-derived growth factor-AA (PDGF-AA), typically present in SASP secretome, could accelerate wound healing in a mouse model of impaired tissue repair [101].

Moreover, inhibitors of genetic pathways involved in senescence-related apoptosis have revealed senolytic activity. For example, it has been shown that the antitumoral agent panobinostat is able to target accumulated senescent cells for their effective clearance. This inhibitor of the histone deacetylases has received FDA approval for the treatment of several malignancies [109].

Similarly, desatinib and quercetin can act in concert to clear senescent adipocyte and endothelial cells in atherosclerotic lesions, by inhibiting the cell death regulator Bcl-2 [110]. BH3 mimetic inhibitors acting on the same gene, as ABT-199, ABT-263, and ABT-737, induce the clearance of senescent HSC and consequently increase the proliferation of the healthy ones [111].

Administration of exogenous IL-10 is particularly beneficial in the clinical treatment of myocardial infarction. In fact, Jung et al. verified that IL-10, an anti-inflammatory cytokine secreted by M2 macrophages, is able to stimulate the monocyte polarization towards the regenerative profile and the activation of fibroblasts, with consequent positive effects on left ventricle function and dilatation [112]. A similar effect can also be achieved by conditioning with activators of SIRT-1 [113]. Analogously, age-related cardiac hypertrophy has been reversed by intraperitoneal injection of recombinant GDF11, a member of the TGF-*β* growth factor superfamily [114].

For the reversal of endothelial cell senescence in atherosclerosis and other pathologies, the administration of a bactericidal/permeability-increasing fold-containing family B member 4 (BPIFB4) isoform has demonstrated to increase the production of NO and stimulates relaxation, as shown by the work of Puca's group [115–119].

Apart from targeting molecular pathways, further strategies to enable senescence protection are represented by the use of different stem cells. Fetal MSC have been confirmed to secrete bioactive factors able to promote proliferation and differentiation in aged MSC [120]. This is particularly relevant in therapies based on adult MSC in order to protect these cells from possible senescence triggers in unfavorable microenvironments. Beneficial effects of MSC secretome are also being exploited in a recent biomedical approach, combining cardiac stem cells and ancillary cellular elements to increase their protection, differentiation, and regeneration [121].

In addition, cardiac stem cells are being infused also in the hostile microenvironment offered by the anthracycline-induced cardiomyopathic heart, whose compromised functions have been ameliorated with this therapeutic approach [113].

We recently described for the first time a significant positive correlation between *β*-blocker (BB) treatment of donor patients and both successful CS isolation and CS-forming cell yield from primary explant cultures. Our results show profound differences in cell phenotype based on their isolation from either BB- or non-*β*-blocker-treated (NBB) patients. In fact, a significantly faster and higher CS-forming capacity was detectable in BB explants compared to NBB. Moreover, an immunophenotypical shift of the described CDC marker CD90 was detectable between the two groups, with a significantly increased percentage of CD90^+^ cells in NBB [122]. In the CADUCEUS clinical trial, CD90 expression in injected CDCs negatively correlated with infarct scar size reduction. This study supports the possible predictive and adjuvant role of *β*-blocker treatment in cardiac cell therapy applications, as recently suggested for mesenchymal stem cell-based therapies. It also suggests novel insights on the influence of BB treatments on the quality and abundance of the cardiac reparative cellular pool [123, 124].

In order to increase the therapeutic success of cardiac stem cell therapies in elderly patients, genetic engineering might be a valid tool too. Avolio et al. have proven that the resveratrol/rapamycin combined treatment of senescent cardiac stem cells derived from decompensated hearts generates an epigenetic cellular reprogramming that reverses the aged phenotype and increases the proliferation [125]. The overexpression of Pim-1 was shown to have analogous effects in senescent cardiac stem cells [126]. The reprogramming approach to pluripotency is a promising powerful strategy to overcome senescence. Yamanaka and his group demonstrated as first the feasibility to convert somatic differentiated cells into induced pluripotent stem cells (iPSCs) by the overexpression of four transcription factors, that is, OCT4, SOX2, c-MYC, and KLF4 [127, 128]. Strikingly, Lapasset et al. induced the pluripotency state in senescent and centenarian fibroblasts, by adding NANOG and LIN28 to the reprogramming gene cocktail used before [129].

Interestingly, the treatment of p16-mediated senescent fibroblasts with inhibitors of BMP-SMAD signaling (e.g., Dorsomorphin, SMAD6, and SMAD7) is able to favor the generation of iPSCs [130].

Lastly, the creation of platforms to model senescence and aging in a tridimensional (3D) tissue environment is of paramount importance to better simulate cell-ECM interactions and develop clinically relevant therapeutic solutions. Recently, two research groups have demonstrated the feasibility to model senescence in 3D settings. Zhou et al. evaluated the effect of oxidative stress on a bioengineered tissue, constructed with a decellularized, MSC-secreted ECM scaffold and human umbilical cord-derived MSC. By comparison to an artificial matrix layer realized with fibronectin and collagen I, the authors showed that cells seeded onto decellularized scaffolds are less incline to develop senescence after H_2_0_2_ stimulation, through an effect mediated by SIRT-1 upregulation [131].

Li et al. reconstructed *in vitro* a 3D cardiac tissue by injecting a mixture of hydrogel, neonatal rat ventricular cardiomyocytes, and fibroblasts in molds of polidymethylsiloxane. They revealed that the age of cardiac fibroblasts is a determining factor in the electrical and mechanical performance of cocultured cardiomyocytes [132], by recapitulating *in vitro* the alterations observed in senescent fibroblasts of the adult heart.

## Figures and Tables

**Table 1:** Senescence in atherosclerotic lesions.

Extrinsic vascular senescence triggers	
(i) High blood cholesterol levels	[17]
(ii) Inflammatory cytokines and growth factors	
(iii) Angiotensin II	
(iv) Hyperglycemia and associated AGEs	

Intrinsic vascular senescence triggers	
(i) Telomere shortening	[17]
(ii) Activation of Ras pathway	
(iii) Mytochondrial dysfunction, mtDNA mutations, and/or release of mitochondria-specific ROS	[18]

Activated (↑) and inactivated (↓) molecular pathways and functions	
(i) Involvement of several genes (e.g., Klotho) and proteins (e.g., Sirtuins, progerin, JunD, p66^shc^, and *β*-amyloid peptides)	[3]
(ii) ↑ DNA damage signaling	
(a) ↑ cyclin-dependent kinase inhibitors (p15, p16, p21, p27, and/or p53)	
(b) ↓ mediators of cell cycle progression (cyclin-dependent kinase inhibitor 2A; cyclin-dependent kinases (1, 2, 4, and 6) and cyclins (A, E, and D))	
(c) ↓ tumor suppressor RB	
(iii) ↑ nuclear factor NF-*κ*B and CCAAT/enhancer-binding protein-*β* pathways	[3, 21]
(iv) ↑ SASP program, which includes the secretion of IL-6, IL-8, chemokines, and activators of macrophages and monocytes (MCP, MIP, TNF-*α*, TGF-*β*, and GM-CSF), as well as ECM proteases	[3, 21]
(v) ↑ procalcific factors (RUNX-2, alkaline phosphatase, collagen I, matrix GLA protein, and BMP-2) in SMCs	[12, 22]
(vi) Impairment of eNOS and prostacyclin pathways in senescent ECs	[14, 24–26]
(vii) Association between decreased levels of CDKN1 and CDKNA2 and increased propensity to develop atherosclerosis	[27, 28]
(viii) Link between CDKN1 and CDKNB2 polymorphisms and aortic aneurism	[29]
(ix) ↑ ICAM-1, PAI-1, and IL-1*α* and ↓ lipid metabolism ability in ECs	[30–33]
(x) ↑ SASP secretion of annexins and BMPs and ↑Ca^2+^ in ECs favors calcification onset	[34]

AGEs: advanced glycosylation end products; SMCs: smooth muscle cells; ECs: endothelial cells.

**Table 2:** Senescence in heart failure.

Extrinsic heart senescence triggers	
(i) Hypercholesterolemia	[33, 36–40]
(ii) Hypertension	

Intrinsic heart senescence triggers	
(i) Leukocyte telomere length shortening	[33, 36–40]

Upregulated (↑) and downregulated (↓) molecular pathways and functions	
(i) ↓ autophagy, mediated by ↑ oxidative stress	
(a) Early signs of senescence, accumulation of dysfunctional mitochondria, disorganization of sarcomere structure, and age-related cardiomyopathy in Atg5-deficient mice	[41]
(b) ↓ cardiac function and survival in Parkin-deficient mice	[42]
(c) Stimulation of mitophagy with spermidine, a natural polyamine, preserves cardiac function in old mice	[43, 44]
(d) Inhibition of miR-22 rescues autophagy and improves cardiac function in old mice	[45, 46]
(ii) Alterations of the inflammatory, endothelial, and myogenic phenotype of cardiac cells are also observed, with most of changes related to the aging-dependent manifestation of mutations in genes involved in the calcium cycling and signaling (↓ activity of SERCA2 and calsequestrin and Ica)	[47, 48]
(iii) ↓ cardiac sympathetic innervation	[49, 50]
(a) ↓ catecholamine uptake, ↓ isoproterenol response, ↓ epinephrine reuptake, and ↓ noradrenalin transport in senescent cardiac neuronal cells	
(iv) Age-dependent hERG mutations	[51]
(a) Disorganization of the structure of the sarcomeric structure and myofibrillary proteins	
(v) ↓ SIRT 3	[52]
(a) Pericyte loss and endothelial dysfunction, further exacerbated in the case of concomitant diabetic condition	
(vi) ↑ ADAM/TACE overexpression in adipose tissue, mediated by ↓ negative regulation by caveolin-1	[53]
(a) ↑ TNF-*α* activity	
(vii) ↑ senescence-associated genetic program in recruited fibroblasts of cardiac ischemia can be considered a protective mechanism from endothelin-1-mediated cardiac fibrosis	[54, 55]

SERCA2: sarcoplasmic reticulum calcium adenosine triphosphatase; Ica: L-type calcium current; hERG: human K^+^ channel ether-à-go-go; SIRT3: mitochondrial nicotinamide adenine nucleotide histone deacetylase.

**Table 3:** Senescence in endogenous stem reservoirs of the heart and in cardiac cell therapy.

CSCs and CDCs	
(i) Signs of senescence	
(a) Reduced telomere length	[80]
(b) Decreased telomerase activity	
(c) Reduced expression of p16^INKA^ and p21^CIP^	
(d) Decreased expression of IL-6- and IGFBP7-enriched SASP	
(e) Increased expression of the Wnt inhibitor Sfrp1and of p16 and a peculiar SASP typical in old subjects (65–83 years), differently from younger ones (2–65 years)	[81]
(ii) Known senescent triggers	
(a) Age-related cardiovascular pathologies, compromising heart function	[83–85]
(b) Antitumoral drugs, as anthracyclines	[82]
(c) Aged animals in physiological settings possess a remarkable number of cardiac stem cells, but dysfunctional in several biological activities, as the metabolism of vitamins and tyrosine, the circadian rhythm, and the complement and coagulation cascades. Consequently, cell proliferation, multipotency, and differentiation abilities are impaired	[86]
(d) Epigenetic modifications	[87]

HSC	
(i) Signs of senescence	
(a) Induced quiescence	[90–93]
(b) Overexpression of Mdr1 and Abcg2 transporters	
(c) Glycolysis-mediated ATP generation	
(d) Telomere shortening	
(e) Accumulated mitochondrial mutations	
(f) Reduced ROS production	
(g) Reduced abilities of self-renewal and differentiation	
(h) Although a high number of HSCs is retrieved in the elderly heart, these undergo a drastic lowering of clonal diversity and switch towards the myeloid lineage, consequently impairing their regenerative abilities	[94]

EPCs	
(i) Signs of senescence	
(a) Overexpressed angiotensin II	[95, 96]
(b) Increased induction of apoptosis	
(c) Reduced levels of SDF-1	
(d) Intensified ROS production	

MSCs	
(i) Signs of senescence	
(a) Classic senescent phenotype	
(b) Reduced migration capacity	[97]
(c) Decreased plasticity	
(d) Alterations in immunoregulatory abilities	[88, 98–100]

CSC: cardiac stem cells; CDCs: cardiosphere-derived cells; HSCs: hematopoietic stem cells; EPCs: endothelial progenitor cells; MSCs: mesenchymal stem cells.

**Table 4:** Current approaches of rejuvenation biotechnology.

Naturally inspired novel drug generations	
(i) rPDGF-AA might accelerate wound healing in a mouse model of impaired tissue repair.	[101]
(ii) FDA-approved antitumoral agent panobinostat is able to target accumulated senescent cells for their effective clearance.	[109]
(iii) Desatinib and quercetin clear senescent adipocyte and endothelial cells in atherosclerotic lesions. BH3 mimetic inhibitors, as ABT-199, ABT-263, and ABT-737, induce the clearance of senescent HSC and consequently increase the proliferation of the healthy ones.	[110, 111]
(iv) Administration of exogenous IL-10 or activators of SIRT-1 is particularly beneficial in the clinical treatment of myocardial infarction, by acting on the macrophages/fibroblast axis.	[112, 113]
(v) Intraperitoneal injection of recombinant GDF11 reverses age-related cardiac hypertrophy.	[114]
(vi) Administration of BPIFB4 increases the production of NO and stimulates relaxation, reversing endothelial cell senescence in atherosclerosis and other pathologies.	[115–119]

Stem cells for senescence protection	
(i) Fetal MSCs secrete bioactive factors promoting proliferation and differentiation in aged MSCs.	[120]
(ii) MSC secretome has beneficial effects in the protection, differentiation, and regeneration of CSCs and ancillary cellular elements.	[121]
(iii) CSCs ameliorate cardiac functionality in the anthracycline-induced cardiomyopathic heart.	[113]
(iv) A significant positive correlation exists between BB treatment of donor patients and both successful CS isolation and CS-forming cells yield from primary explant cultures. A significantly faster and higher CS-forming capacity was detectable in BB explants compared to NBB. A significantly increased percentage of CD90^+^ cells was observed in NBB CDCs.	[122]
(v) CD90 expression in injected CDCs negatively correlated with infarct scar size reduction (CADUCEUS trial). This study supports the possible predictive and adjuvant role of *β*-blocker treatment in cardiac cell therapy applications, as recently suggested for MSC-based therapies. It also suggests novel insights on the influence of BB treatments on the quality and abundance of the cardiac reparative cellular pool.	[123, 124]

Stem cell engineering and reprogramming strategies	
(i) Resveratrol/rapamycin induces an epigenetic cellular reprogramming in senescent CSCs derived from decompensated hearts, by increasing cell proliferation.	[125]
(ii) PIM overexpression in senescent CSCs reverses heart aging.	[126]
(iii) Pluripotency reprogramming is feasible in centenarian cardiac fibroblasts by OCT4, SOX2, c-MYC, KLF4, NANOG, and LIN28 overexpression.	[129]
(iv) Treatment with inhibitors of BMP-SMAD signaling, for example, Dorsomorphin, SMAD6, and SMAD7, generates iPSCs in p16-mediated senescent fibroblasts.	[130]

In vitro 3D tissue engineering platforms to model senescence and aging	
(i) Evaluation of the effect of oxidative stress on a bioengineered tissue, constructed with a decellularized, MSC-secreted ECM scaffold and human umbilical cord-derived MSC. By comparison to an artificial matrix layer in fibronectin and collagen I, cells seeded onto decellularized scaffolds are less incline to develop senescence after H_2_0_2_ stimulation, through an effect mediated by SIRT-1 upregulation.	[131]
(ii) Age of cardiac fibroblasts is a determining factor in the electrical and mechanical performance of cocultured cardiomyocytes in an *in vitro* 3D cardiac tissue, composed of a mixture of hydrogel and fibroblasts in molds of polidymethylsiloxane. This model is useful to recapitulate *in vitro* the alterations observed in senescent fibroblasts of the adult heart.	[132]

rPDGF-AA: recombinant platelet-derived growth factor-AA; BPIFB4: bactericidal/permeability-increasing fold-containing family B member 4; MSCs: mesenchymal stem cells; CSCs: cardiac stem cells; CSs: cardiospheres; BB: *β*-blocker; NBB: non-*β*-blocker; iPSCs: induced pluripotent stem cells; ECM: extracellular matrix.
